# Continental scale comparison of mycobiomes in *Parmelia* and *Peltigera* lichens from Turkey and South Korea

**DOI:** 10.1186/s12866-024-03388-0

**Published:** 2024-07-04

**Authors:** Jiho Yang, Jung-Jae Woo, Cenk Sesal, Barış Gökalsın, Vahap Eldem, Birkan Açıkgöz, Tunahan Irmak Başaran, Gamze Kurtuluş, Jae-Seoun Hur

**Affiliations:** 1 Korean Lichen Research Institute, Sunchon National University, 255 Jungang‑ro, Suncheon, South Korea; 2 Department of Biology, Marmara University, Istanbul, TR-34722 Turkey

**Keywords:** Lichen, Mycobiome, Community structure, Diversity, Turkey, South Korea

## Abstract

**Background:**

Lichens, traditionally considered as a simple partnership primarily between mycobiont and photobiont, are, in reality, complex holobionts comprised of a multitude of microorganisms. Lichen mycobiome represents fungal community residing within lichen thalli. While it is acknowledged that factors like the host lichen species and environmental conditions influence the structure of the lichen mycobiome, the existing research remains insufficient. To investigate which factor, host genus or location, has a greater impact on the lichen mycobiome, we conducted a comparative analysis of mycobiomes within *Parmelia* and *Peltigera* collected from both Turkey and South Korea, using high-throughput sequencing based on internal transcribed spacer region amplification.

**Results:**

Overall, the lichen mycobiome was dominated by Capnodiales (Dothideomycetes), regardless of host or location. At the order level, the taxonomic composition was not significantly different according to lichen genus host or geographical distance. Hierarchical clustering of the top 100 abundant ASVs did not clearly indicate whether the lichen mycobiome was more influenced by host genus or location. Analyses of community similarity and partitioning variables revealed that the structure of the lichen mycobiome is more significantly influenced by location than by host genus. When analyzing the core mycobiome by host genus, the *Peltigera* mycobiome contained more ASV members than the *Parmelia* mycobiome. These two core mycobiomes also share common fungal strains, including basidiomycete yeast. Additionally, we used chi-squared tests to identify host genus-specialists and location-specialists.

**Conclusions:**

By comparing lichen mycobiomes of the same genera across different countries, our study advances our comprehension of these microbial communities. Our study elucidates that, although host species play a contributory role, geographic distance exerts a more pronounced impact on the structure of lichen mycobiome. We have made foundational contributions to understanding the lichen mycobiome occupying ecologically crucial niches. We anticipate that broader global-scale investigations into the fungal community structures will provide more detailed insights into fungal residents within lichens.

**Supplementary Information:**

The online version contains supplementary material available at 10.1186/s12866-024-03388-0.

## Introduction

Lichens represent a symbiotic organism between mycobiont and photobiont including green algae and/or cyanobacteria [1]. Approximately 400–600 million years ago, lichen symbiosis evolved [2], with the fungal component acting as the structural scaffold for the lichen thallus, offering both a sheltered habitat for the photosynthetic partner and a conduit for nutrient exchange [3, 4]. Remarkably adaptable, lichens can inhabit a broad spectrum of terrestrial environments, spanning from arid deserts to polar regions [5, 6], and have gained recognition as essential bioindicators of environmental dynamics, manifesting sensitivity to alterations in air quality and fluctuations in climate [7, 8]. Lichens are integral to pivotal ecological processes, notably nutrient cycling and nitrogen fixation [9–11]. They exert notable influence in both biophysical and biochemical weathering processes, contributing significantly to plant succession and the development of soil [12]. This underscores the importance of delving into the intricate ecological roles of lichens to enhance our comprehensive comprehension of terrestrial ecosystems.

Lichens, traditionally viewed as a simple partnership primarily between fungi and algae, are in fact intricate ecosystems composed of a myriad of microorganisms [13]. Lichen mycobiome, denoting fungi residing within lichen thalli, comprises diverse taxa [14]. They have been discovered in virtually all lichen species investigated thus far, representing an array of taxonomic groups, with many yet to be formally described [15]. Consequently, lichen mycobiome constitute a relatively underexplored facet of fungal diversity, sparking interest for their capacity to produce secondary metabolites endowed with diverse biological activities [16]. The precise ecological role of these entities within lichen thallus remains not fully elucidated. Nevertheless, it is conjectured that their diverse bioactive compounds may contribute to functions akin to endophytes, such as nutrient cycling and enhancement of host immunity [14, 15]. Consequently, delving into the intricacies of lichen mycobiome is not only an exercise in uncovering their species diversity and distribution but is also crucial for unveiling their ecological functions and interactions. This knowledge stands to bolster the promotion of sustainable ecosystems and their associated ecological benefits. Multiple factors, encompassing the host lichen species and environmental conditions can exert influence on the structure of lichen mycobiome [14, 15, 17, 18].

Recently, Yang et al. revealed that, within South Korea, the lichen mycobiome is more profoundly shaped by geographical variations rather than the lichen host haplotype [19]. To gain a broader perspective, we expanded our investigation to an international scale. We conducted a comparative analysis of mycobiomes within *Parmelia* and *Peltigera* lichen species collected from similar geographical locations in both Turkey and South Korea based on high-throughput sequencing with internal transcribed spacer region amplification. Our analysis of the lichen mycobiome centered on three specific inquiries: First, we aimed to understand the overall taxonomic diversity within the mycobiome. Second, we sought to determine whether host phylogeny or geographic distance had a more pronounced influence on the mycobiome structure. Lastly, we aimed to identify the core members and specialists within the mycobiome, guided by the factors revealed in our investigation. We posit that unraveling the processes governing structure of the lichen mycobiome, even across considerable geographical distances between nations, holds merit in our research endeavors.

## Methods

### Lichen sampling

In the year 2022, we conducted lichen collection at two distinct locations: Uludag Mountain in Turkey’s Bursa Province and Hanla Mountain in Jeju Island (South Korea). We gathered samples of both *Parmelia* and *Peltigera* species, collecting four samples each of *Parmelia* and *Peltigera* from both the Turkish and South Korean sites (Table S1). These 16 lichen thalli were further subdivided into four pieces each to facilitate bioreplication testing, resulting in a total of 64 samples for subsequent analysis. After collection, the samples were delicately placed into collection bags and transported to the laboratory. They were subsequently stored in an ultra-low temperature freezer until further experiments were conducted.

Morphological identification of each lichen sample was performed directly in the field, and subsequent molecular identification was carried out based on the internal transcribed spacer (ITS) region in our laboratory. The lichen thalli, obtained during this study, were dissected into 1 cm² pieces, with any surface residues removed through a combination of syringe tip extraction and thorough washing with running tap water. Following this initial preparation, the lichen thalli surfaces were sterilized using a solution of 70% ethanol and 0.4% sodium hypochlorite according to Yang’s protocol [20], with the sterilization process lasting for 90 s. This method was employed to isolate fungi from lichens, but we anticipate that the running tap and ethanol treatment processes might help remove some of the DNA from other fungi present on the lichen surface. Furthermore, even if a small amount of epiphytic fungal DNA is included in the subsequent data, we believe it will be negligible compared to the portion occupied by the lichen mycobiome DNA.

### Molecular and bioinformatics analyses

The lichen segments underwent homogenization through bead lysis, and their DNA was subsequently extracted using the PowerSoil DNA isolation kit (QIAGEN, CA, USA). By amplifying the ITS region of the extracted DNA, we conducted a phylogeny test for the host lichen. The polymerase chain reaction (PCR) conditions were as follows: an initial denaturation step at 94 °C for 5 min, followed by 35 cycles of 94 °C for 30 s, 55 °C for 30 s, and 72 °C for 40 s, with a final extension at 72 °C for 10 min. We entrusted Macrogen (Seoul, South Korea) with Sanger sequencing to obtain the ITS sequences amplified with primers ITS4 and ITS5 [21], which were subsequently aligned using the MAFFT ver. 7 [22]. The information for the lichen sequences used in the host phylogeny has been included in Table S2. Through a phylogenetic model test conducted with MEGA ver. 7 [23], we confirmed that our samples were best suited to the Tamura’s 3-parameter + G + I model [24]. Based on this model, we obtained a maximum likelihood [25] phylogeny with 1000 bootstrap iterations. The visualization of host lichen phylogeny was conducted using iTOL [26].

We proceeded with PCR targeting the fungal ITS1 region [14] for metabarcoding. We employed primers ITS1F and ITS2 [27], incorporating Illumina sequencing adaptors. PCR was carried out in triplicate for each sample, utilizing the AccuPower PCR PreMix kit (Bioneer, Daejeon, South Korea), under the following conditions: an initial denaturation step at 94 °C for 5 min, followed by 25 cycles of 94 °C for 30 s, 55 °C for 30 s, and 72 °C for 40 s, with a final extension at 72 °C for 10 min. The quality of the PCR products was assessed using agarose gel electrophoresis, followed by purification with the Expin PCR SV kit (GeneAll, Seoul, South Korea). A second PCR was conducted for barcoding, incorporating multiple index delimiters as per the Nextera XT Index kit protocol (Illumina, CA, USA). Amplicon concentration was determined using the NanoDrop2000 (Thermo Fisher Scientific, MA, USA), and PCR products were pooled in equal molecular quantities. Subsequently, amplicon library sequencing was executed on an Illumina MiSeq platform by Macrogen (Seoul, South Korea).

Raw sequences underwent initial processing using QIIME2 ver. 2021.4 [28], including demultiplexing and denoising reads following the DADA2 pipeline [29]. Taxonomic assignment was executed in accordance with the Naïve Bayesian classifier guidelines [30], utilizing the UNITE fungi 99% OTU database [31]. A phylogenetic analysis was conducted employing the q2-alignment plugin based on RAxML [32]. All sequencing data were deposited in the NCBI, Sequence Read Archive, under the accession number PRJNA1035977. The amplicon sequence variant (ASV) [33] table was imported from QIIME2 to R using the ‘qiime2R’ package (https://github.com/jbisanz/qiime2R). Host lichen sequences were filtered out, and the ASV table was rarefied to 1,800 sequences per lichen host for comparison in equal standard using the ‘phyloseq’ package [34]. In this procedure, ten samples were omitted. Subsequent analyses were conducted using the rarefied ASV table.

### Statistics and visualization

To begin the statistical comparisons, we initially applied the Shapiro test [35] to assess data normality. Given the violation of normality assumptions, non-parametric tests were employed, including the Mann–Whitney U test [36] for pairwise comparisons and the Kruskal-Wallis test [37] followed by the Bonferroni correction [38] for multiple comparisons. All subsequent analyses were conducted using R software version 3.5.3 [39]. Visualization of all graphs was carried out using the ‘ggplot2’ package [40]. For the creation of hierarchical heatmap depicting the distribution of the top 100 abundant ASVs, the ‘pheatmap’ package was employed [41]. To showcase the two-dimensional distribution of the lichen mycobiome, we utilized the ‘microbiome’ package for canonical correspondence analysis ordination [42]. Variation partitioning and nestedness visualization were generated using the ‘vegan’ package [43]. Identification of the core mycobiome was accomplished using the ‘microbiome’ package, and specialist testing was conducted by employing linear discriminant analysis based on the ‘microbial’ package.

## Results

### Lichen host phylogeny

Phylogenetic analysis based on ITS sequence data revealed that all the samples we gathered belong to the genera *Parmelia* and *Peltigera* (Supplementary Fig. S1). The *Parmelia* samples collected in Turkey represented a single species, *P. submontana*, while the *Parmelia* samples from Korea were identified as *P. praesquarrosa*. Our *Peltigera* samples were shown to encompass a total of four distinct species. The *Peltigera* species from Turkey included *Peltigera* aff. *neocanina*, *Peltigera* aff. *membranacea*, *Pe. degenii*, and *Pe. praetextata*. In contrast, all *Peltigera* samples from Korea were identified as *Pe. degenii*. In summary, *Parmelia* samples from Turkey and Korea each represented a single species, while the *Peltigera* samples collected in Turkey showed greater species diversity compared to those from Korea.

### Taxonomic diversity of lichen mycobiome

**Fig. 1:**
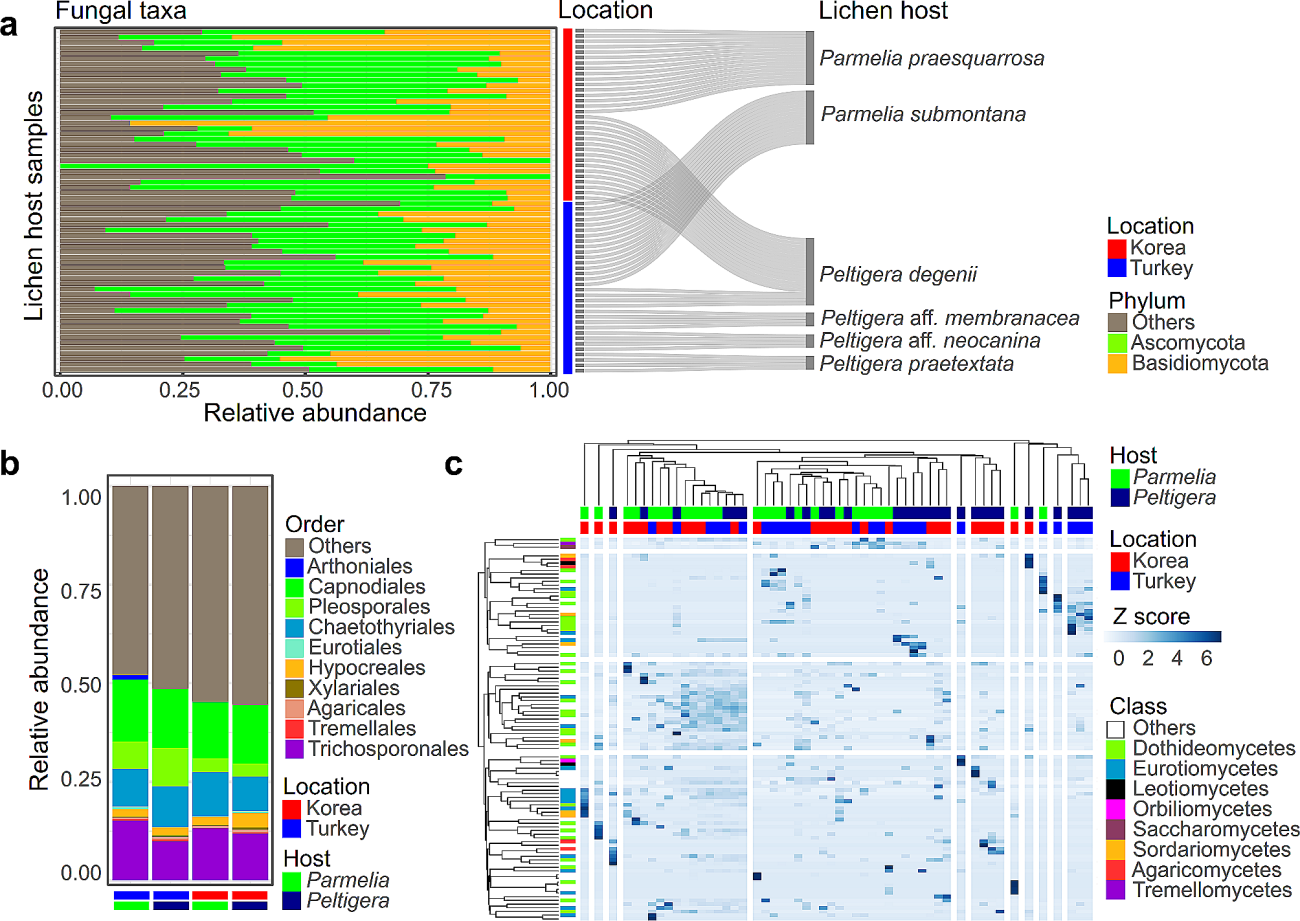
Taxonomic diversity of the lichen mycobiota The taxonomic composition of the lichen mycobiome was visualized using a stacked bar plot at the phylum level (a). The taxonomic composition at the order level was presented for the four lichen host types (b). Color keys representing location and host were provided below the bar plot. A hierarchical heatmap of the top 100 most abundant ASV members was arranged based on abundance similarity (c). ASV abundances were standardized using Z-scores, and the top two rows at the top of the heatmap were color-coded to represent host and location. The taxa to which ASVs belong were indicated on the left column, color-coded accordingly

In order to assess the overall diversity of the lichen mycobiome, we observed the taxonomic composition (Fig. 1a). At the phylum level, except for unclassified fungi, members of Ascomycota (40%) were the most abundant, with fungi belonging to Basidiomycota (24%) following. We investigated whether the taxonomic composition of the lichen mycobiome varied based on host type at the order level (Fig. 1b). There were no significant differences in diversity observed among the four host types: Korean *Parmelia* and *Peltigera*, and Turkish *Parmelia* and *Peltigera*. When compared across countries, the proportion of Pleosporales was higher in the Turkish lichen mycobiome (8%) than Korean’s (3%), regardless of host species.

To understand the factors influencing the clustering of the lichen mycobiome, we investigated a hierarchical heatmap of the top 100 abundant ASVs based on host lichen genus and geographical differences (Fig. 1c). It was not clear which of these two factors had a greater impact on the clustering. This suggests that additional analysis is needed to clearly identify the factors shaping the lichen mycobiome. To summarize, it can be concluded that the lichen mycobiome, in terms of taxonomic diversity, does not exhibit significant differentiation based on host phylogeny or geographical distance, and it appears to share similar features.

### Influential factors in shaping the lichen mycobiome and alpha diversity of the mycobiome

**Fig. 2:**
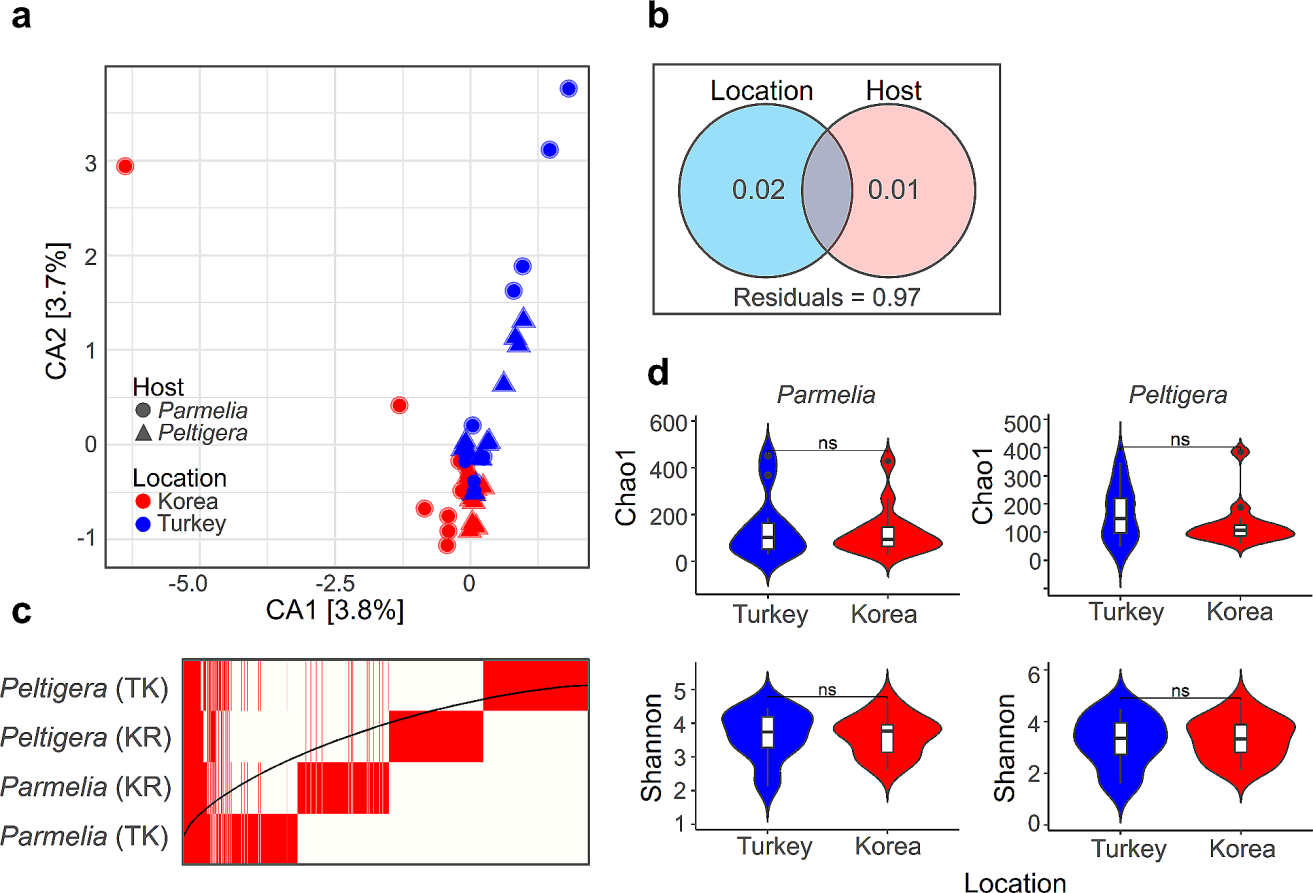
Factors shaping the lichen mycobiome and alpha diversity comparison The lichen mycobiome similarity was ordinated based on Bray-Curtis distance through canonical correspondence analysis (a). The numerical values indicated on the x and y-axes represent the explanatory power of the ordination axes, with host genus and location represented by different shapes and colors. Variation partitioning was presented in a Venn diagram format (b). The explanatory values were rounded to two decimal places. A graph displaying nestedness based on the four host types was plotted, with the x-axis representing ASV lists and the y-axis indicating host types (c). Red color represented presence, white indicated absence, and as the number of measured ASVs increased, diversity was rarified. Alpha diversity was visualized using a violin plot (d). ^ns^*p* > 0.05 (Mann-Whitney U test)

To explore which factor, either the host genus or geographical distance, has a more significant impact on the lichen mycobiome, we conducted a two-dimensional ordination through canonical correspondence analysis (Fig. 2a). It appears that the lichen mycobiome structure is more influenced by location similarity. However, we believed that further statistical testing was necessary and thus performed a variation partitioning analysis (Fig. 2b). While the size of the residual component was substantial (0.97), it conclusively revealed that the lichen mycobiome is more strongly influenced by location (0.02) than by the host (0.01).

We also examined how alpha diversity of the lichen mycobiome varies according to host genus and locational difference. Nestedness is a term used when one group exhibits a subset-like pattern within another group, and it is useful for assessing overall alpha diversity. As shown in the figure, regardless of location, *Peltigera* mycobiome has a more nested structure compared to fungal community inside *Parmelia* (Fig. 2c). For Chao1, representing species richness, there were no significant differences between *Parmelia* and *Peltigera* mycobiomes across locations (Mann-Whitney U test, *p* > 0.05, Fig. 2d). Similarly, Shannon index, which signifies species diversity, did not exhibit significant differences based on location (Mann-Whitney U test, *p* > 0.05). Additionally, there were no notable distinctions in Chao1 and Shannon index when comparing lichen mycobiomes based on their host genera *Parmelia* and *Peltigera* (Mann-Whitney U test, *p* > 0.05, Supplementary Fig. S2a). In summary, the lichen mycobiome is more strongly influenced by location differences than by the host genus, and *Peltigera* mycobiome appears to have a more nested structure than that of *Parmelia*.

### Core members of the lichen mycobiome

**Fig. 3:**
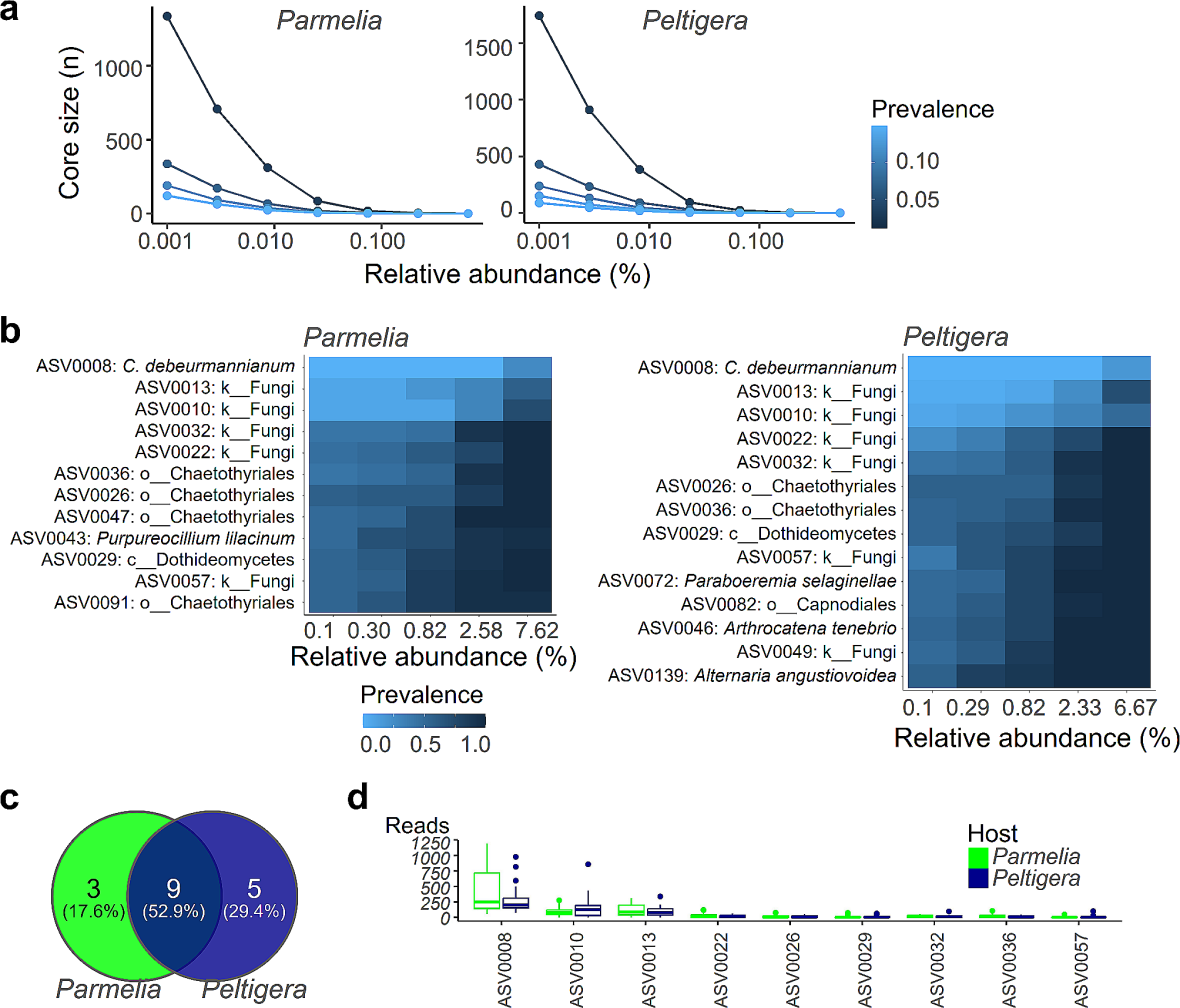
Core members in the lichen mycobiome The core size (the number of core members) was determined by two variables, relative abundance, and prevalence, and the relationship between these variables is depicted in a line plot (a). We used a relative abundance cutoff of 0.1%, and the heatmap visualizes the prevalence of core mycobiome members based on their relative abundance (b). The common part of the two core mycobiomes was represented using a Venn diagram (c). The number of sequence reads in the common core mycobiome was depicted using a box plot (d)

Generally, the term ‘core microbiome’ refers to a set of members that are commonly found within different hosts or samples. In other words, the core mycobiome signifies the fungal species that appear consistently, regardless of the habitat. We aimed to identify the core mycobiomes within the two genera, *Parmelia* and *Peltigera*, utilizing two measures: relative abundance and prevalence (Fig. 3a). As the cutoff values for both measures increased, the number of core members (core size) decreased. When we set the relative abundance cutoff to 0.1%, we discovered 12 core members for *Parmelia* and 14 core members for *Peltigera* (Fig. 3b). Interestingly, among the core mycobiomes of the two different lichen hosts, there were several common fungal taxa, such as *Cutaneotrichosporon debeurmannianum*, Chaetothyriales, and Dothideomycetes (Fig. 3c). Among them, the abundance of *C. debeurmannianum* was relatively high (Fig. 3d). In summary, we have identified the core mycobiomes of *Parmelia* and *Peltigera*, and these two core mycobiomes share common fungal ASVs.

### Host or locational specialist in lichen mycobiome

**Fig. 4:**
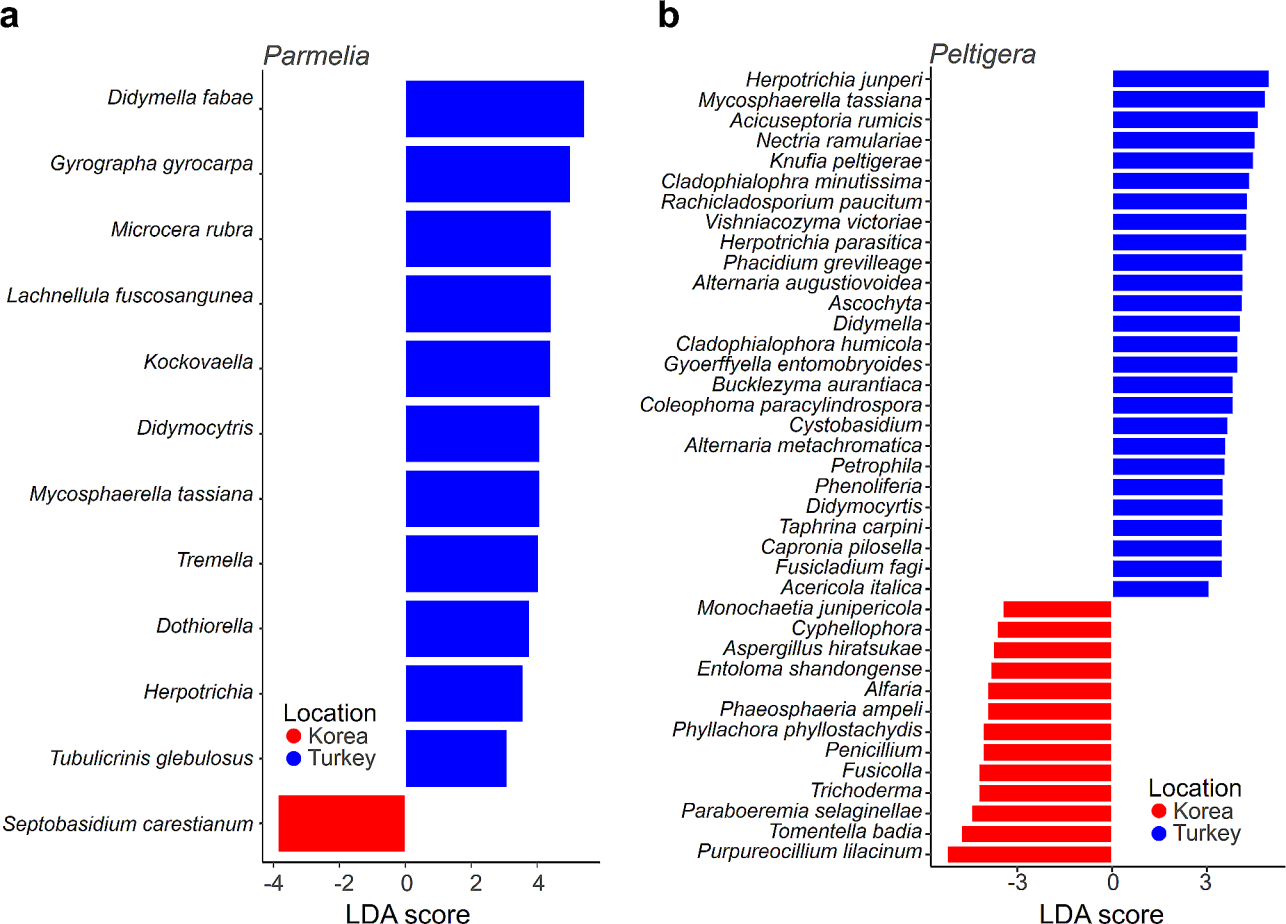
Specialized residents in the lichen mycobiota The regional specialists of *Parmelia* (a) and *Peltagera* (b) mycobiomes were visualized through linear discriminant analysis. The displayed taxa included only those identified up to the genus level with an LDA score of 3.0 or higher

Linear discriminant analysis (LDA) is a useful tool in the field of microbial community analysis for identifying microbial taxa with statistically significant differences in abundance. In our study, we employed LDA to identify host genus or location-specific specialists. We focused on data that were identified at the genus level and had LDA scores of 3.0 or higher, excluding unclassified data such as unclassified family data. In the *Parmelia* mycobiome, we observed the frequent presence of eleven taxa, including *Didymella fabae* and *Gyrographa gyrocarpa* in lichens from Turkey (Fig. 4a). Conversely, in Korean *Parmelia*, we found that only *Septobasidium carestianum* was a specialist. In the *Peltigera* mycobiome, we confirmed the significant abundance of sixteen taxonomic groups, including *Herpotrichia junperi* and *Mycosphaerella tassina*, in Turkish samples (Fig. 4b). Additionally, thirteen taxonomic groups, including *Monochaetia junipericola* and *Cyphellophora*, were significantly abundant in Korean *Peltigera*.

We also investigated lichen mycobiome specialists at the host genus level (Supplementary Fig. S2b). Among *Parmelia* specialists, three taxonomic groups, including *Gyrographa gyrocarpa*, were identified, while in *Peltigera*, 32 unique taxonomic groups, including *Phlebia*, were significantly more abundant as specialists. In summary, our comparison between Turkish and Korean lichens revealed a higher number of specialists in Turkish lichen communities, and when comparing lichen genera, *Peltigera* exhibited a greater number of specialists than *Parmelia*.

## Discussion

In this study, we embarked on an exploration of lichen mycobiomes, aiming to unravel their taxonomic diversity, the influential factors governing their composition, and the identification of core members and specialists. Our findings shed light on several intriguing aspects of lichen mycobiomes and their ecological relevance.

The examination of the lichen mycobiome’s taxonomic diversity revealed intriguing insights into the fungal communities within lichens. At the phylum level, the dominance of Ascomycota, followed by Basidiomycota, is consistent with previous studies [17, 19]. The prevalence of Dothideomycetes, Eurotiomycetes, Tremellomycetes, and at the class level showcases the importance of these fungal groups in lichen mycobiomes. The orders Capnodiales within Dothideomycetes and Chaetothyriales belonging to Eurotiomycetes have been recognized as major constituents of lichen mycobiomes in previous studies and are part of the endolichenic or lichenicolous groups, and they have been shown not to be transient residents [44]. These groups consist mainly of slow-growing fungi and were not frequently isolated or illuminated when attempting to culture them from lichen samples. However, with the advancement of metabarcoding technology, they have gained significant attention [44]. This fungal group is characterized by a high degree of melanization, a feature that is likely associated with their exceptionally slow growth [45, 46]. Fungi that produce melanized cell walls tend to have a sluggish growth rate [47]. As a result, we posit that these fungi exhibit resilience in the harsh lichen microenvironment and are able to sustain a relatively high abundance. Notably, the taxonomic composition did not exhibit significant differentiation based on host phylogeny or geographical distance, indicating shared features within lichen mycobiomes across diverse host genera and locations.

An intriguing aspect of our study was the investigation of the factors influencing the composition of lichen mycobiomes. Through a two-dimensional ordination and a variation partitioning analysis, we discerned that geographical location had a more substantial impact on the lichen mycobiome than the host genus. This observation aligns with recent research, which also highlighted the significance of geographic variations in shaping lichen mycobiomes [14, 19]. The finding of shared lichenicolous fungi among taxonomically diverse lichens within the same habitat further corroborates the earlier discoveries [44]. Geographical variations, including climate, elevation, and local microenvironments, can influence the availability of fungal partners and resources, thereby driving the differentiation of lichen mycobiomes. Further exploration of the specific environmental parameters that underlie these geographical differences is essential for a more comprehensive understanding of lichen mycobiome ecology.

The identification of core members and specialists within lichen mycobiomes adds depth to our understanding of the key constituents in these ecosystems. The presence of common fungal ASVs within the core mycobiomes of *Parmelia* and *Peltigera*, despite different host genera and geographical locations, suggests the existence of functional groups that transcend these boundaries. The presence of common core members, such as *Cutaneotrichosporon debeurmannianum*, Chaetothyriales, and Dothideomycetes, underscores their adaptability and versatility in lichen symbioses. The prevalence of Dothideomycetes and Eurotiomycetes within lichen communities, as previously described, has been well-documented in several studies [17, 19, 44]. However, the unexpected discovery of *C. debeurmannianum* as a core member of lichen communities in this study is noteworthy. This fungus, which has previously been isolated as a rare basidiomycete yeast, has been found in clinical samples, including cases of diabetic foot infections [48, 49]. Previously, Spribille’s research garnered attention by molecularly demonstrating the presence of basidiomycete yeasts in various lichens [50]. However, these taxa belong to the genus *Cystobasidium* are phylogenetically distant from the *Cutaneotrichosporon* identified in this study. There is a need for further research to elucidate the ecological niche of this fungus within the lichen thallus. Our exploration of specialists within the lichen mycobiome unveiled notable differences between Turkish and Korean lichens, as well as disparities between *Parmelia* and *Peltigera*. Understanding the functions of these specialists and their interactions with lichen hosts remains an intriguing avenue for future research. Notably, the greater prevalence of core members and host-specific specialists in *Peltigera* suggests that the mycobiome of *Peltigera* demonstrates a more nested structure in comparison to *Parmelia*. This assertion is substantiated by the findings of nestedness analysis.

Our study attempted to compare the lichen mycobiomes of Turkey and Korea using various analytical methods, but it has some limitations. Dividing a single lichen thallus into four pieces for analysis may be perceived as pseudoreplication. However, this approach also helps account for intra-community dissimilarity. Previous studies have shown that the structure of the lichen mycobiome can vary within different parts of the thallus, indicating the necessity of sample replication within thalli [18, 20]. Although this study compares lichen mycobiomes on a continental scale, the collection of only two lichen genera from two mountains is insufficient to support broad conclusions. A more extensive and dense selection of sites, as well as the inclusion of a wider variety of lichen taxa, is needed. Our sampling was not sufficient to compare various species within a single host lichen genus, and within our limited sampling scope, the microbiomes of different lichen species within a single genus were not significantly different. This is likely because the types of secondary metabolites secreted by different species within the genus *Parmelia* do not vary significantly (this also applies to *Peltigera*) [51, 52]. Previous findings that reported no significant differences in bacterial community composition between two *Endocarpon* species support our results [53]. There was a high proportion of unclassified fungi within the lichen mycobiome [17, 19, 54]. The study of lichen mycobiomes is relatively recent, so it is likely that our samples contain various species not yet registered in the sequence database, possibly including new species. This could explain the high proportion of unclassified fungi, a characteristic noted in previous research as well. Future researchers studying lichen mycobiomes should consider contributing to the sequence database by discovering new species and carefully deciding how to handle unclassified fungal sequences when analyzing mycobiome structures. In the analysis of lichen mycobiome beta diversity, the explanatory power of partitioning variables and CA scores were observed to be very low. This suggests that, in addition to the variables we set (lichen host genus and location difference), there are many other factors influencing the mycobiome. Incorporating quantitative variables such as climatic data, along with the qualitative variables, could provide a more detailed understanding of the factors affecting the structure of the lichen mycobiome.

Our findings have important implications for the ecology of lichen mycobiomes. The consistent taxonomic diversity across host genera and geographic locations suggests a level of adaptability and resilience in lichen mycobiomes. This adaptability may be pivotal for their role as bioindicators, as they can withstand variations in environmental conditions and reflect ecological changes through their mycobiome composition. Furthermore, the prevalence of geographical influence on lichen mycobiome composition highlights the need to consider regional variations when studying and conserving lichen communities. To promote the sustainable functioning of ecosystems, it is crucial to acknowledge the pivotal role of lichen mycobiomes in nutrient cycling, carbon fixation, and environmental resilience.

## Conclusions

By cross-country comparisons of lichen mycobiomes within the same genera, our research enhances our understanding of these microbial communities. We uncovered their taxonomic diversity, the factors that shape their composition, and the core members and specialists within these intricate ecosystems. Our findings clarify that, while host genus contribute, geographic distance exerts a more significant influence on the structure of lichen mycobiome. Furthermore, our study contributes foundational insights into the understanding of lichen mycobiomes occupying ecologically vital niches. We anticipate that comprehensive global-scale investigations into lichen mycobiome structures will offer more detailed insights into the fungal residents within lichens.

### Electronic supplementary material

Below is the link to the electronic supplementary material.

Supplementary Material 1

## Data Availability

All sequencing data are accessible on Sequence Read Archive (PRJNA1035977), NCBI.

## Abbreviations

ASV: amplicon sequence variant
ITS: internal transcribed spacer
LDA: Linear discriminant analysis
PCR: polymerase chain reaction (PCR)
