# Supplementary material for: Continental scale comparison of mycobiomes in Parmelia and Peltigera lichens from Turkey and South Korea
Source: BMC Microbiol. 2024 Jul 4;24:243. doi: 10.1186/s12866-024-03388-0 (PMC11223332; doi:10.1186/s12866-024-03388-0)
Supplement: Supplementary file 1 — Supplementary Material 1 [file 12866_2024_3388_MOESM1_ESM.docx]

**Additional file 1**

**Table S1**. Collection information of host lichens

| **Sample** | **Accession** | **Location** | **GPS** | **Altitude (m)** | **Substrate** | **Species** |
| --- | --- | --- | --- | --- | --- | --- |
| KR01 | PP832228 | Korea | 33°23'33.01" N, 126°29'45.29" E | 966 | bark | *Parmelia praesquarrosa* |
| KR02 | PP832229 | Korea | 33°23'34.02" N, 126°29'44.74" E | 960 | bark | *Parmelia praesquarrosa* |
| KR03 | PP832230 | Korea | 33°23'34.02" N, 126°29'44.74" E | 960 | bark | *Parmelia praesquarrosa* |
| KR04 | PP832231 | Korea | 33°23'31.06" N, 126°29'45.04" E | 971 | bark | *Parmelia praesquarrosa* |
| KR05 | PP832232 | Korea | 33°23'52.09" N, 126°27'36.72" E | 686 | rock | *Peltigera degenii* |
| KR06 | PP832233 | Korea | 33°23'52.09" N, 126°27'36.72" E | 686 | rock | *Peltigera degenii* |
| KR07 | PP832234 | Korea | 33°23'52.09" N, 126°27'36.72" E | 686 | rock | *Peltigera degenii* |
| KR08 | PP832235 | Korea | 33°23'52.09" N, 126°27'36.72" E | 686 | rock | *Peltigera degenii* |
| TK09 | PP832236 | Turkey | 40°06'09.82" N, 029°17'15.68" E | 1525 | bark | *Parmelia submontana* |
| TK10 | PP832237 | Turkey | 40°06'10.06" N, 029°17'15.77" E | 1519 | bark | *Parmelia submontana* |
| TK11 | PP832238 | Turkey | 40°09'09.74" N, 029°17'16.04" E | 1517 | bark | *Parmelia submontana* |
| TK12 | PP832239 | Turkey | 40°06'00.41" N, 029°16'59.58" E | 1652 | bark | *Parmelia submontana* |
| TK13 | PP832240 | Turkey | 40°06'00.73" N, 029°16'59.79" E | 1650 | rock | *Peltigera degenii* |
| TK14 | PP832241 | Turkey | 40°06'00.38" N, 029°16'58.28" E | 1651 | soil | *Peltigera* aff*. membranacea* |
| TK15 | PP832242 | Turkey | 40°06'03.17" N, 029°17'11.91" E | 1560 | rock | *Peltigera* aff*. neocanina* |
| TK16 | PP832243 | Turkey | 40°06'42.82" N, 029°19'08.47" E | 978 | soil | *Peltigera praetextata* |

**Table S2**. The information of the reference sequences from GenBank used in the host phylogeny

| **No.** | **Lichen species** | **Accession no.** |  |  |
| --- | --- | --- | --- | --- |
| 1 | *Parmelia adaugescens* | AY036991 | AY036992 | KM250147 |
| 2 | *Pa. barrenoae* | AY295103 | AY579446 | MW793515 |
| 3 | *Pa. cochleata* | AY036985 | AY036986 |  |
| 4 | *Pa. crambidiocarpa* | GU994571 |  |  |
| 5 | *Pa. cunninghamii* | GU994572 |  |  |
| 6 | *Pa. discordans* | AY583212 |  |  |
| 7 | *Pa. encryptata* | AY579456 | MZ557930 | OR509532 |
| 8 | *Pa. ernstiae* | AF350041 | MT581504 | OR509536 |
| 9 | *Pa. erumpens* | AY037001 |  |  |
| 10 | *Pa. fertilis* | AY036982 | KM250182 | KT625496 |
| 11 | *Pa. fraudans* | KT625499 | KT625500 | MK812511 |
| 12 | *Pa. homogenes* | KY128188 | KY128189 |  |
| 13 | *Pa. hygrophila* | JN609436 | KT625508 | KT625509 |
| 14 | *Pa. imbricaria* | KT625503 | KT625507 | NR_148085 |
| 15 | *Pa. laevior* | AY036995 | DQ394387 | DQ394388 |
| 16 | *Pa. marmariza* | KM250179 |  |  |
| 17 | *Pa. mayi* | JN118585 |  |  |
| 18 | *Pa. mayi* | JN118586 |  |  |
| 19 | *Pa. mayi* | JN118585 | JN118586 | JN118587 |
| 20 | *Pa. omphalodes* | AY036998 | HQ026238 | KP226214 |
| 21 | *Pa. pinnatifida* | AY036988 | MN412774 | MN412791 |
| 22 | *Pa. praesquarrosa* | KT625510 | LC533078 |  |
| 23 | *Pa. pseudolaevior* | DQ394389 |  |  |
| 24 | *Pa. saxatilis* | AF350024 | AF350034 | AY036990 |
| 25 | *Pa. serrana* | AF350031 | AY036997 | AY295109 |
| 26 | *Pa. shinanoana* | KM250186 | KM250188 | KM250189 |
| 27 | *Pa. signifera* | AY037003 |  |  |
| 28 | *Pa. skultii* | AY251456 | FJ425881 | OR500513 |
| 29 | *Pa. squarrosa* | AY036976 | AY036979 | HQ026234 |
| 30 | *Pa. subdivaricata* | KM250175 | KM250176 | KM250178 |
| 31 | *Pa. submontana* | AY037000 | MG676386 | MZ558017 |
| 32 | *Pa. sulcata* | AY580313 | EU788027 | MW793518 |
| 33 | *Pa. sulymae* | KT625527 | NR_148086 |  |
| 34 | *Peltigera aphthosa* | AF158645 | FJ708820 | U73492 |
| 35 | *Pe. austroamericana* | MH758438 | MH758456 | MH758457 |
| 36 | *Pe. britannica* | AF158646 | FJ708846 | U73493 |
| 37 | *Pe. canina* | AY257952 | FJ708873 | FJ708890 |
| 38 | *Pe. collina* | AF074976 | AY257969 | FJ708923 |
| 39 | *Pe. degenii* | AY257901 | FJ709030 | GQ292456 |
| 40 | *Pe. didactyla* | AF350295 | AY266027 | FJ527258 |
| 41 | *Pe. dolichorrhiza* | AF350293 | FJ527279 | JX195206 |
| 42 | *Pe. extenuata* | AY257937 | AY257939 | JX195275 |
| 43 | *Pe. fuscopraetextata* | AY257911 | FJ708893 | MH758501 |
| 44 | *Pe. gowardii* | JF837354 | JF837359 | JF837360 |
| 45 | *Pe. horizontalis* | AY257959 | FJ708930 | KC437645 |
| 46 | *Pe. hydrothyria* | JF837365 | KM005783 | KM005789 |
| 47 | *Pe. hymenina* | JX195208 | KC139754 | KX897191 |
| 48 | *Pe. kristinssonii* | AY257891 | FJ708935 | FJ708949 |
| 49 | *Pe. leucophlebia* | AF158651 | FJ708954 | U73494 |
| 50 | *Pe. malacea* | AY257965 | FJ708859 | U73491 |
| 51 | *Pe. membranacea* | AY257906 | AY257907 | FJ709031 |
| 52 | *Pe. monticola* | AY257872 | KC139753 | KJ413221 |
| 53 | *Pe. neocanina* | AY257954 | AY257955 | FJ708916 |
| 54 | *Pe. neopolydactyla* | AF350292 | FJ709036 | GQ292460 |
| 55 | *Pe. neorufescens* | AY257916 | KJ413246 | KJ616384 |
| 56 | *Pe. occidentalis* | KX897268 | MF908489 | MG811760 |
| 57 | *Pe. patagonica* | MH758226 | MH758228 | OP602866 |
| 58 | *Pe. polydactyloides* | AY257967 | FJ527275 | MK517835 |
| 59 | *Pe. polydactylon* | GQ292455 | GQ292464 | KC437643 |
| 60 | *Pe. ponojensis* | AY257883 | FJ709040 | KF957622 |
| 61 | *Pe. praetextata* | AY257900 | DQ001296 | FJ708904 |
| 62 | *Pe. rufescens* | AY257925 | JX195332 | KJ413220 |
| 63 | *Pe. rufescentiformis* | MH758372 | MH758373 | MZ385659 |
| 64 | *Pe. scabrosa* | JX195222 | KX897301 | KX897316 |
| 65 | *Pe. scabrosella* | KX897328 | KX897329 | MK812406 |
| 66 | *Pe. seneca* | KX365450 | KX365451 | MZ385677 |
| 67 | *Pe. ulcerata* | AY257957 | FJ527259 | KF913003 |


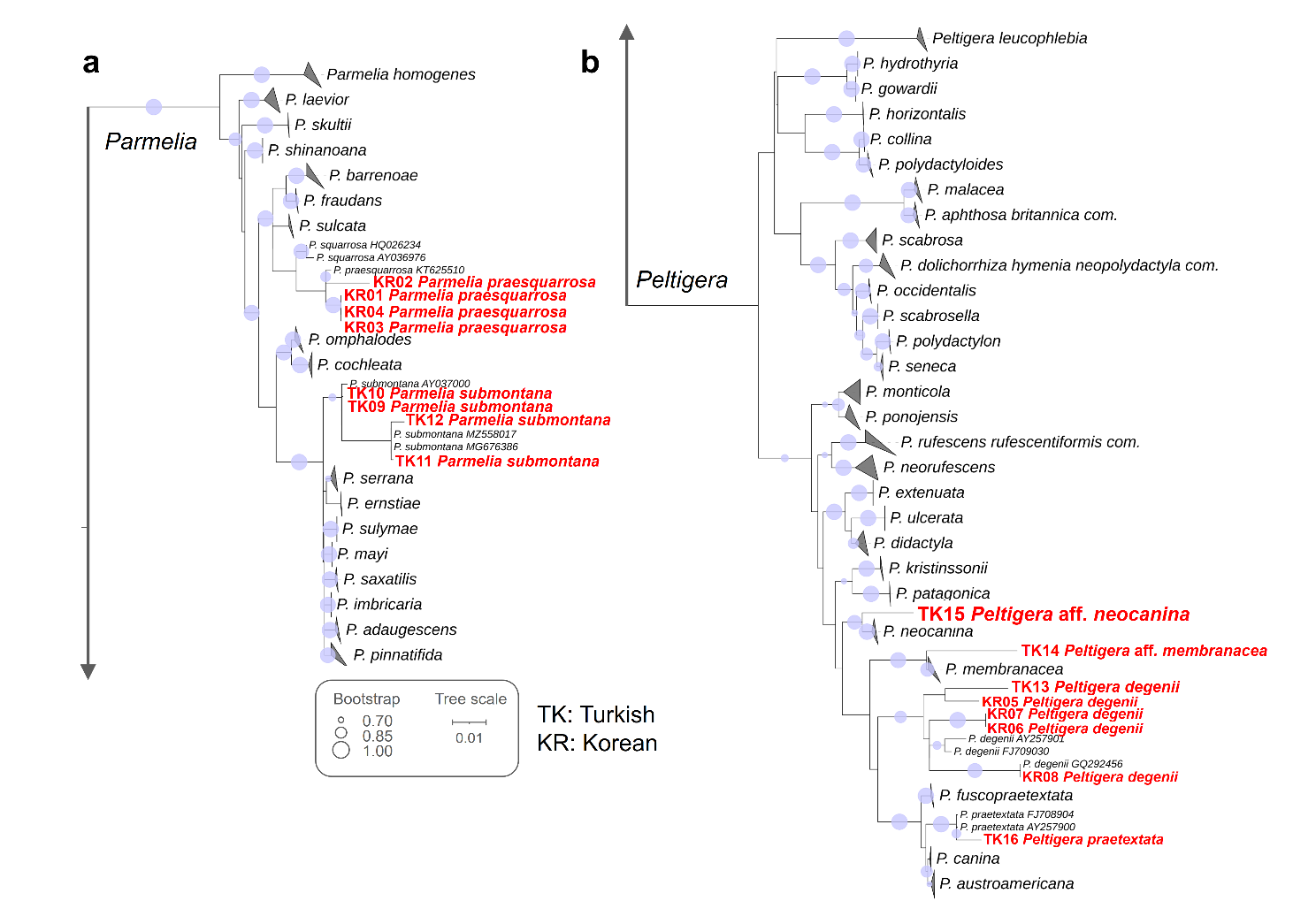


**Supplementary figure S1**. Lichen host phylogeny

The lichen hosts *Parmelia* (a) and *Peltigera* (b) were identified to the species level using the internal transcribed spacer region. The phylogenetic tree was constructed using the Tamura 3-parameter + G + I model, which was selected after model testing, and the maximum likelihood method. Bootstrap values from 1,000 repetitions were visualized with circle sizes for values greater than 70%.


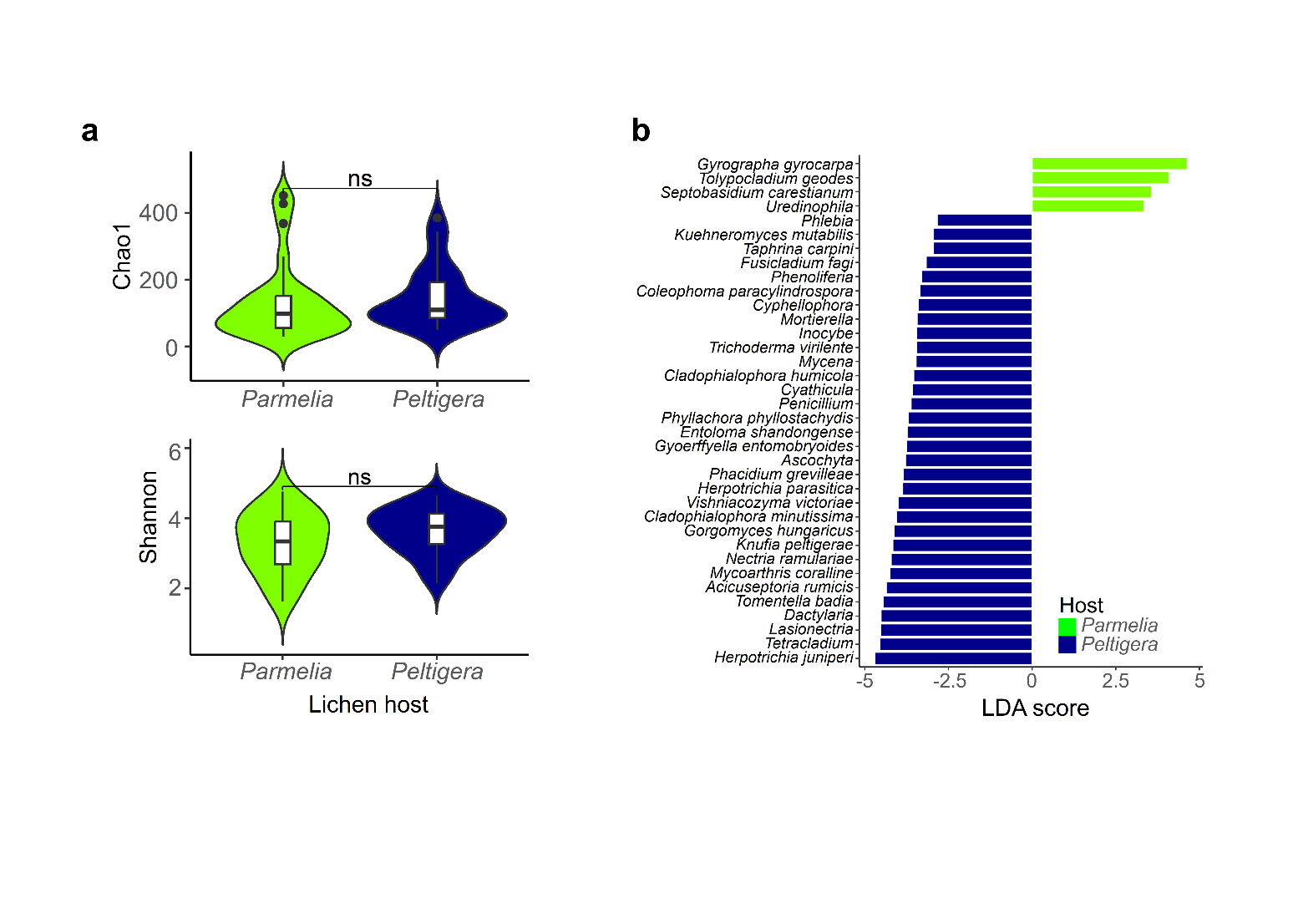


**Supplementary figure S2**. Alpha diversity comparisons and a specialist list based on the host genus

Species richness represented by Chao1 and species diversity represented by the Shannon index were visualized using violin plots (a). Host specialists of the *Parmelia* and *Peltagera* mycobiomes were depicted through linear discriminant analysis (b). The taxa displayed consisted of only those identified up to the genus level with an LDA score of 3.0 or higher.
